# Farm management practices and season dependent factors affect the microbial community and chemical profile of corn and grass-legume silages of farms in Ontario, Québec, and Northern New York

**DOI:** 10.3389/fmicb.2023.1214915

**Published:** 2023-07-19

**Authors:** Jesse Huffman, Pascal Drouin, Justin B. Renaud, Lysiane Dunière, Gisèle LaPointe

**Affiliations:** ^1^Department of Food Science, Dairy at Guelph, Ontario Agricultural College, University of Guelph, Guelph, ON, Canada; ^2^Independent Researcher, Saint-Jean-sur-Richelieu, QC, Canada; ^3^Agriculture and Agri-Food Canada, Southern Crop Protection and Food Research Center, Ottawa, ON, Canada; ^4^Lallemand SAS, Animal Nutrition, Blagnac, France

**Keywords:** silage, inoculant, corn, legume, metabolites, mycotoxins, bacteria, fungi

## Abstract

The effects of farm management practices and seasonal variation on the microbial community and chemical composition of corn and grass-legume silage are largely understudied due to the advantages of controlled mini-silo experiments. This study aims to investigate the effects that some key farm factors (use of an inoculant, farm region, and bunker or tower silo) and seasonal variations have on corn and grass-legume silage from farms across Ontario, Quebec, and New York. The silage was either treated with a commercial inoculant (Lallemand Biotal Buchneri 500® or Chr Hansen SiloSolve FC®) or left untreated. The bacterial communities of silage were compared to those of raw bulk tank milk from the same farm to determine if they were similarly affected by management practices or seasonal variations. Family level analysis of the 16S rRNA V3-V4 gene amplicon bacterial community, the ITS1 amplicon fungal community, NMR water soluble metabolome, and mycotoxin LC–MS were performed on silage over a two-year period. Chemical compounds associated with the use of inoculants in corn and grass-legume silage were higher in inoculated corn (acetate, propane-1,2-diol, γ-aminobutyrate; *p* < 0.001) and grass-legume (propionate; *p* = 0.011). However, there was no significant difference in the relative abundance (RA) of *Lactobacillaceae* in either silage type. *Leuconostocaceae* was higher in non-inoculated corn (*p* < 0.001) and grass-legume (*p* < 0.001) silage than in inoculated silage. Tower silos had higher RA of *Leuconostocaceae* (*p* < 0.001) and higher pH (*p* < 0.001) in corn and grass-legume silage. The one farm that used liquid manure with no other fertilizer type had higher RA of *Clostridiaceae* (*p* = 0.045) and other rumen/fecal (*p* < 0.006) bacteria in grass-legume silage than all other farms. Seasonal variation affected most of the key silage microbial families, however the trends were rarely visible across both years. Few trends in microbial variation could be observed in both silage and bulk tank milk: two farms had higher *Moraxellaceae* (*p* < 0.001) in milk and either corn or grass-legume silage. In farms using an inoculant, lower *Staphylococcaceae* was observed in the raw bulk tank milk.

## 1. Introduction

Corn, grasses, and legumes are common forage crops used as dairy cattle feed across the globe, and ensiling is the most common form of feed preservation. The temperature variation throughout the year with a particular impact of the cold season in the northern regions of North America, Europe, and Asia highlight the importance of silage preservation (Kennang Ouamba et al., 2022). Epiphytic bacteria from the field forage, particularly lactic acid bacteria (LAB), will often dominate the microbial community of ensiled forage. Those LAB drive silage quality and the presence of potentially harmful microorganisms both to cattle health and dairy processing (Borreani et al., 2018; Driehuis et al., 2018). Epiphytic LAB can drop the pH of silage rapidly within the first week of ensiling (Borreani et al., 2018). However, depending on the specific LAB present in the silage and conditions of fermentation (such as forage buffering capacity, dry matter at ensiling, available sugar, packing density, among others) the pH decrease may be slow, leaving time for other epiphytic bacteria to compete such as *Enterobacteriaceae* (Borreani et al., 2018), *Clostridiaceae* (Li et al., 2020), *Bacillaceae*, and potentially pathogenic bacteria (Ávila and Carvalho, 2020).

The use of LAB as silage inoculants is common on dairy farms throughout North America to increase aerobic stability, preserve forage, increase feed efficiency, and even improve milk production when feeding common forage crops such as grasses, legumes, and corn (Muck et al., 2018). Inoculants are often a combination of obligate homofermentative strains responsible for rapid pH drop through lactic acid production (*Pediococcus pentosaceus*, *Pediococcus acidilactici*, *Enterococcus faecium*, *Lactococcus lactis*), facultative heterofermentative strains (*Lactiplantibacillus plantarum*), and obligate heterofermentative strains (*Lentilactobacillus buchneri*, *Lentilactobacillus hilgardii*, *Lentilactobacillus diolivorans*) of LAB producing organics acids with antifungal properties and enhancing aerobic stability (Weinberg and Muck, 1996). Their effects on the microbiota of silage and aerobic deterioration have been shown in mini-silo operations to be effective at controlling epiphytic bacteria and fungi (Drouin et al., 2021).

In controlled silage experiments such as mini-silos, exposure to air, humidity, and temperature changes are intentionally controlled (Kim and Adesogan, 2006), so single factors can be explored to measure individual effects. This is not the case in large scale silage operations on farms, where silage fermentation conditions vary from farm to farm depending on specific conditions such as silo type, forage growing conditions, packing density, ensiling temperature, storage periods, regional considerations, and individual practices (Filya et al., 2007; Muck et al., 2015; Windle and Kung, 2016; Randby et al., 2020). However, because of the nature of silage research and the use of mini silos, there is little to no information on most of these specific effects, due to the difficulties in studying these factors experimentally in commercial operations.

The purpose of this study was to determine the effects of seasonal factors (temperature) and farm specific factors (epiphytic bacteria, fertilizers) on the microbial community and metabolomic composition of corn and mixed grass-legume silage in commercial management systems on seven farms spanning Ontario, Québec, and northern New York state. Furthermore, the samples were divided by silo type (varying in density and exposure to air) and the use of an inoculant or not (impacting pH drop and acetic acid content), to determine if these factors were able to affect any of the microbial variations linked to season of feed-out and geographical location.

## 2. Materials and methods

### 2.1. On-site farm sampling

The seven farms included 2 farms in Southwestern Ontario, Canada, 4 farms in the Montérégie region, Québec, Canada, and 1 farm in Clinton County in northeastern New York state, USA who accepted to participate in this study. Of the seven farms, three stored the silage in tower silos and four stored the silage in bunker silos. Four farms used an inoculant (INO; two bunkers, two towers), and three farms did not use a microbial additive on the forage (NIS; two bunkers, one tower; Table 1). Farms L05, Q02, and Q03 used Biotal Buchneri 500® (*Pediococcus pentosaceus* 12455 and *Lentilactobacillus buchneri* 40788) as inoculant for the corn and the grass-legume silage, while farm E01 used Chr Hansen SiloSolve FC® (*Lentilactobacillus buchneri* LB1819 and *Lactococcus lactis* O224). Samples of corn and mixed grass-legume forage and silage as well as the milk were taken every 2 months for 2 years starting in September of 2018 and ending in July of 2020. Sampling of the silage was performed as soon as possible after the morning preparation of the total mixed ration on the farms. The sampling procedure of bunker silos was to remove 5 cm of the face layer that was exposed to oxygen and sample around 500 grams of silage, collected from five different points across the bunker face and then pooled into one sample. This procedure was done three times on each sampling day, for a total of three pooled samples per silage type per sampling day. For tower silos, the feed was run for 30 s to clear silage that had been exposed to oxygen, and then around 500 grams of sample were taken directly from the feed chute three times. A total of 42 samples of grass-legume and 42 samples of corn per farm were collected and analyzed for a total of 252 grass-legume and 252 corn silage samples across both years.

**Table 1:** Information on the management practices of the seven participating farms.

	Farms						
	L01	L05	Q01	Q02	Q03	Q04	E01
Location	Southwestern	Southwestern	Montérégie	Montérégie	Montérégie	Montérégie	Clinton
Province or State	Ontario	Ontario	Québec	Québec	Québec	Québec	New York
Microbial additive	NIS	INO	NIS	INO	INO	NIS	INO
Silo type	Bunker	Tower	Tower	Bunker	Tower	Tower	Bunker
Fertilizer applied							
Grass-alfalfa	LM + MI	LM + MI	MI	MI	MI	LM + MI	LM
Period of application	EC	EC	EC	EC	SP + EC	EC	EC
Corn	SM + MI	LM + MI	SM + MI	SM + MI	SM + MI	LM + MI	SM + MI
Forage composition							
Timothy	X	X			X	X	
Brome grass	X	X	X	X	X	X	
Canary grass							X
Orchard grass	X						X
Fescue		X	X			X	
Festulolium				X			
Alfalfa			X	X	X	X	X
Clover				X	X		
% grasses over legumes	60%	20%	55%	60%	65%	60%	70%
Corn							
Conventional	Yes	Yes	Yes	Yes	Yes	Yes	Yes
BMR	No	No	No	No	No	No	Yes
% BMR in TMR							30%
Number of cattle	~240	~60	~110	~130	~50	~45	~600
Housing	Free-stall	Free-stall	Free-stall	Free-stall	Tie-stall	Tie-stall	Free-stall
Milking system	Parallel	Robot	Robot	Parallel	In stall	In stall	Parallel

Microbial additives: INO: microbial additives inoculated on forage at harvest; NIS: no microbial additives applied on forage. Fertilizer: [SM] solid manure; [LM] liquid manure; [MI] mineral. Fertilizer period of application: [SP] spring; [EC] after each cut; [MS] mid-growing season; [LC] after last cut. Milking: [Parallel] Manual system in milking parlor; [In stall] Manual milking in stall; [Robot] Automatic milking. BMR: brown mid- corn hybrid. TMR: total mixed ration of the dairy cattle.

Fresh forage of mixed grass-legume and corn was taken in triplicate from the forage truck on-farm directly after cutting at 2 cm theoretical length of cut. There were three cuts of mixed grass-legume forage per year (spring, summer, fall), and one cut of corn per year (fall; Table 1). Total samples collected and analyzed from forage harvests were 18 for grass-legume per farm (total of 126 samples for grass-legume forage across both years) and 6 for corn per farm (total of 42 samples for corn forage across both years). All samples were placed on ice for transportation to the laboratory and placed into a − 20°C freezer until further analysis.

Milk sampling was done in single replicate at each silage sampling period across 2 years for a total of 12 milk samples per farm and 84 for all farms across both years. Samples were taken by stirring the bulk milk tank for 30 s before running a small amount of milk into the drain from the bottom tank valve and then placing a 200 ml container under the running milk. All samples were placed on ice for transportation to the laboratory and placed into a −20°C freezer until further analysis.

### 2.2. DNA extraction and 16S rRNA gene and ITS amplicon sequencing

DNA extraction for the forage and silage samples was performed according to the methodology described by Drouin et al. (2019). Overall, 5 g of sample was placed into a 50 ml conical tube and filled with 10 ml of sterile water. Tubes were then placed in a sonicator bath (FB-11203, Thermo Fisher, Waltham, MA, USA) and sonicated for 10 min at 50°C, after which they were vortexed for 1 min at high speed. Three milliliters of supernatant were then transferred to two sterile microcentrifuge tubes while avoiding as much plant debris as possible. Microtubes were centrifuged at 20.65 × *g* for 30 s to precipitate any large plant debris in the supernatant. Avoiding the pellet containing plant debris, the supernatant was then transferred to a clean microtube and centrifuged for 1 min at 10,000 × *g*. The supernatant was removed, and the pellets were treated using the protocol provided in the *Quick*-DNA Fecal/Soil Microbe Miniprep DNA extraction kit (Zymo Research, Irvine, CA, USA).

Preparation of the milk samples for DNA extraction was performed according to the methodology described by Julien et al. (2008). A 1.5 ml solution of 25% w/v sodium citrate was heated up to 45°C and mixed with 25 ml of raw milk in a stomacher bag. The bag was then homogenized in a stomacher at 200 rpm for 5 min, and the contents were then transferred to a 50 ml conical tube. The tube was centrifuged at 15,000 × *g* for 15 min. The supernatant and cream were poured out of the falcon tube and fat was removed with a cotton swab, leaving a cell pellet. The cell pellet was then washed three times by suspending in 1 ml of 2% sodium citrate using a pipette and centrifuged at 10,000 × *g* for 5 min before removing the supernatant. The cell pellet was then treated using the protocol provided in the *Quick*-DNA Fecal/Soil Microbe Miniprep DNA extraction kit (Zymo Research, Irvine, CA, USA).

DNA concentration was measured using Qubit Fluorometric Quantitation (Qubit 4, Invitrogen, Waltham, MA, USA) and then diluted to 5 ng/ml and placed into 96-well microplates. Two sets of samples were then submitted to the University of Guelph Genomics Facility for library preparation and sequencing of the 16S V3-V4 rRNA gene region for bacterial amplicons and the ITS1 region for fungal amplicons using the Illumina Miseq platform. Briefly, the 16S V3-V4 rRNA and ITS1 regions were amplified separately using a limited cycle standard PCR. Primers used for the initial amplification of the 16S amplicons were S-D-Bact-0341-b-S-17 (5′-TCG TCG GCA GCG TCA GAT GTG TAT AAG AGA CAG CCT ACG GGN GGC WGC AG) and S-D-Bact-0785-a-A-21 (5′-GTC TCG TGG GCT CGG AGA TGT GTA TAA GAG ACA GGA CTA CHV GGG TAT CTA ATC C) to produce amplicons under 550 bp (Klindworth et al., 2013). For the ITS1, initial amplification of a set of 8 forward primers and 7 reverse primers were pooled to ensure that gaps in taxonomic coverage that may be present with the use of a single ITS1 amplification primer set were covered (Bellemain et al., 2010; Deshpande et al., 2016). ITS1 amplification was designed to amplify regions usually ranging from 145 and 695 bp. The PCR products were then ligated with Illumina flow cell adapters, gene specific adapters, and index primers to prepare for sequencing. Universal flow cell adapter sequences were forward (5′-AAT GAT ACG GCG ACC ACC GAG ATC TAC AC) and reverse (5’-CAA GCA GAA GAC GGC ATA CGA GAT). Gene specific adapter sequences were forward (5’-TCG TCG GCA GCG TC) and reverse (5’-GTC TCG TGG GCT CGG). There were 8 unique forward index primers and 12 unique reverse index primers to cover all 96 samples in each MiSeq run.

### 2.3. Quantification of water-soluble chemical compounds

Water soluble chemical compounds were quantified using nuclear magnetic resonance at the University of Guelph NMR Center. One hundred and eighty milliliters of distilled water were added to 20 g of mixed grass-legume or corn silage in a blender and mixed for 1 min. Using a large funnel with gauze, the blended sample was poured into a 250 ml Erlenmeyer flask. After the liquid was filtered with 4 × 4 medical gauze pads (AMD Medicom Inc., Montréal, QC, Canada), 50 ml was moved to a conical tube and the pH was read using a pH meter. The remainder of the liquid was further filtered using Grade 1 circular filter paper (Whatman plc, Maidstone, UK) into a 15 ml conical tube. The filtered liquid was then further filtered into microcentrifuge tubes using 0.22 mm syringe filters (MilliporeSigma, Burlington, MA, USA). A final volume of 630 μl was then mixed thoroughly with 70 μl of ChenomX DSS NMR internal standard (Chenomx, Edmonton, AB, Canada) and submitted to the NMR center of the University of Guelph for analysis.

Chenomx NMR Mixture Analysis software was used to measure the molar concentration of peaks of 43 of the most common compounds found across silage samples identified using the Chenomx proprietary reference database. Molar concentrations were then converted into grams/kilogram of dry matter.

### 2.4. Mycotoxin analysis

The multi-mycotoxin method of Sulyok et al. (2006) was used to extract mycotoxins from forage and silage samples. Briefly, 0.2 ± 0.02 g of silage ground to 1 mm was extracted with 1.3 ml of 79/20/1 (v/v/v) acetonitrile/water/acetic acid. The solutions were first vortexed for 30 s, sonicated at 35°C for 30 min, and shaken on a thermomixer (35°C, 1400 rpm) for 30 min. The samples were then centrifuged and 140 μl were removed. The 140 μl extracts were diluted in 60 μl of water and placed at 4°C for 30 min. The samples were centrifuged, and the supernatants transferred to 250 μl polypropylene vials prior to LC–MS/MS analysis. The samples were analyzed using a targeted, muti-residue MS/MS method, analyzed by a Thermo Vanquish Duo Tandem UHPLC System coupled to a TSQ Altis triple quadrupole mass spectrometer (Thermo Fisher Scientific). Samples were stored in an autosampler at 10°C, and 5 μl was injected onto a Zorbax Eclipse Plus RRHD C18 column (2.1 × 100 mm, 1.8 μm; Agilent) maintained at 35°C with a flow rate of 300 μl/min. Mobile phase A (Optima LC–MS grade water +0.1% formic acid) was held at 98% for 1.0 min. Subsequently, mobile phase B (Optima LC–MS grade acetonitrile +0.1% formic acid) was increased to 22% over 0.25 min, then increased to 35% over 2.75 min. Mobile phase B was increased again to 100% over 3.5 min and held for 2.5 min before returning to 2% over 0.5 min. The OptaMax NG H-ESI source was operated with capillary voltage of 3.5 kV in positive mode and 3.25 kV in negative mode, an ion transfer tube temperature of 325°C, and vaporizer temperature of 295°C. The sheath, auxiliary, and sweep gases were set to 25, 20, and 1 arbitrary units, respectively. The Altis mass spectrometer monitored the transitions with a Q1 and Q3 resolution of 0.7 and 1.2 FWHM resolution, respectively. The argon collision gas was maintained at 1.5 mTorr in the collision cell.

### 2.5. Bioinformatics analysis

For taxonomic classification of 16S V3-V4 rRNA gene amplicons in corn, grass-legume, and milk, raw Miseq data were run through the phyloseq DADA2 pipeline (filtering, denoising forward and reverse reads, merging forward and reverse reads, and removing chimeras) and rarefaction curves were calculated. The majority of samples adequately covered the microbial community based on rarefaction curves at over 5,600 reads. Samples were then normalized to the samples with the lowest remaining read count. Taxonomy was classified using the Silva 136 database for 16S amplicons and UNITE for ITS amplicons. Data was agglomerated to family level and filtered based on a 0.1% relative abundance threshold present in at least 15% of samples to account for rare taxa unique to a single farm. Sampling periods were grouped into seasons by combining September/November as Fall, January/March as Winter, and May/July as Summer.

Alpha and beta diversity were calculated using the phyloseq pipeline using filtered ASV tables and classified by silo type, inoculation, farm, and season. Standard alpha diversity metrics for microbial community analysis were performed (Shannon, Simpson, Chao1) and Shannon was chosen as the most relevant index in this study, as it considers both presence/absence and abundance of taxa. Weighted and unweighted UniFrac and Bray–Curtis dissimilarity were used to report beta diversity indices.

### 2.6. Sparse partial least square discriminant analysis and correlations

Family level taxonomic data generated from the phyloseq pipeline as well as NMR water soluble compound quantification were submitted to the sPLS-DA pipeline of the mixOmics package in R.^1^ The sPLS-DA for all factors were tuned to 2 components after running 10-fold cross-validation 100 times using perf and keepX functions to determine the lowest classification error rate for each factor. sPLS-DA were then plotted separately by either 16S rRNA gene amplicons or NMR compounds using components 1 and 2, and contributions to the axes were plotted to determine families and NMR compounds with significant values within factors (significance was calculated as described in the following Statistical Analysis section). Correlation plots between bacterial families and physicochemical parameters (pH and dry matter) for corn and grass-legume silage were generated using the corplot package in R.

### 2.7. Statistical analysis

All statistical significance was calculated using either Wilcoxon for two variable comparisons or Kruskall-Wallis one-way analysis of variance for multi-variable comparisons using the FSA package in R by either silo type, inoculation, farm, or season. Forage samples were separated by forage type and statistical analysis was done by farm only. Kruskal-Wallis one-way analysis of variance supplemented with Dunn’s test for post-hoc pairwise comparisons was performed on each individual variable.

## 3. Results

### 3.1. Bacterial diversity of the forage

At harvest, the relative abundance (RA) of the 10 main bacterial families and four bacterial family groupings (plant-based, rumen-based, facultative anaerobic spore formers, and potentially pathogenic) in the corn ranged between 44.14 and 90.14% (Table 2). RA of *Moraxellaceae* was higher in forage from farm L05 than from all other farms. RA of *Rhizobiaceae* was significantly higher in farms E01 and Q04 than in farm L01 (*p* = 0.008). RA of endophytic bacteria (*p* = 0.005) was significantly higher in forage from farm E01 and Q04 than L01 and L05. There were no other significant differences between farms in family level RA in fresh corn forage, but trends for higher *Leuconostocaceae* and *Enterobacteriaceae* RA were observed in forage from farm L01 while trends for higher *Lactobacillaceae* and *Pseudomonadaceae* RA were noted in forage from farms L05 and Q03, respectively.

**Table 2:** Relative abundance (%) of the most common bacterial families from the 16S rRNA gene amplicon sequencing of DNA extracted from the freshly cut corn.

Bacterial families	Farms (Relative abundance in % and *p* values)							
	E01	L01	L05	Q01	Q02	Q03	Q04	*p*
*Leuconostocaceae*	0.22^1^	12.97	0.71	4.90	0.12	0.92	2.49	0.079^2^
*Lactobacillaceae*	0.94	0.16	1.32	45.15	1.67	2.51	1.66	0.062
*Acetobacteriaceae*	0.42	0.14	0.22	0.18	0.23	0.42	0.63	0.619
*Clostridiaceae*	0.10	0.03	0.04	0.03	0.22	0.11	0.07	0.873
*Enterobacteriaceae*	12.11	67.01	19.02	12.36	17.67	23.88	18.29	0.079
*Rhizobiaceae*	14.11a	0.87b	5.19ab	4.96ab	9.59ab	10.36ab	15.24a	0.008
*Moraxellaceae*	0.40ab	0.85ab	4.54a	0.10b	0.74ab	0.81ab	0.81ab	0.005
*Pseudomonadaceae*	5.30	5.45	4.55	4.70	7.49	17.18	8.77	0.053
*Caryophanaceae*	< 0.01	< 0.01	< 0.01	< 0.01	< 0.01	< 0.01	0.01	0.374
*Streptococcaceae*	0.34	0.49	0.10	0.41	0.23	0.29	0.21	0.186
Facultative anaerobic spore-formers^3^	0.40	0.01	0.29	0.26	1.16	0.77	0.85	0.121
Endophyte bacteria^4^	24.26a	1.72b	2.97b	7.39ab	20.66a	13.52ab	17.06a	0.005
Rumen/fecal bacteria^5^	0.59	< 0.01	0.49	0.42	0.29	0.37	0.30	0.103
Potentially pathogenic bacteria^6^	0.02	0.42	4.69	0.01	0.03	0.22	0.33	0.412

1 Mean relative abundance of 16S V3-V4 amplicon taxonomic data of the top 12 most common bacterial families in corn forage.

2 *p* values were generated by Kruskal-Wallis one-way non-parametric test at an alpha value of 0.05 and post-hoc pairwise comparisons were carried out with Dunn’s test. Lowercase letters represent comparison between farms where the same letter denotes a non-significant difference.

3 Facultative Anaerobic Spore Formers: *Bacillaceae, Paenibacillaceae.*

4 Endophytic bacteria: *Sphingomonadaceae, Sphingobacteriaceae, Xanthomonadaceae.*

5 Rumen/Fecal bacteria: *Lachnospiraceae, Ruminococcaceae.*

6 ‘Potentially pathogenic bacteria’: *Listeriaceae, Staphylococcaceae*.

The RA of the 10 main bacterial families and four groups in mixed grass-legume forage ranged from 51.96 to 71.89% (Table 3). Relative abundance of *Lactobacillaceae* in mixed grass-legume forage was significantly higher in farm Q02 (*p* < 0.001). RA of *Caryophanaceae* (formerly *Planococcaceae*) was significantly higher in farm Q01 than all other farms, except L05 (*p* = 0.045). RA of *Pseudomonadaceae* was significantly lower in forage from farms E01, Q02, and Q04 than all other farms except farm Q03 (*p* = 0.007). There were no other significant differences in family level RA between farms in fresh mixed grass-legume forage except a trend for a lower RA of *Enterobacteriaceae* in forage from the Q1 farm.

**Table 3:** Relative abundance (%) of the most common bacterial families from the 16S rRNA gene amplicon sequencing of DNA extracted from the freshly cut grass-legume forage.

Bacterial families	Farms (Relative abundance in % and *p* values)							
	E01	L01	L05	Q01	Q02	Q03	Q04	*p*
*Leuconostocaceae*	1.72^1^	5.12	0.05	0.76	8.40	0.49	2.01	0.354^2^
*Lactobacillaceae*	0.17a	0.26a	0.44abc	0.35ab	4.92c	1.05bc	4.31ab	<0.001
*Acetobacteriaceae*	0.05	0.12	0.10	0.14	0.33	0.10	0.10	0.448
*Clostridiaceae*	0.02	0.02	0.00	0.41	0.09	0.00	0.05	0.158
*Enterobacteriaceae*	46.68	33.85	36.17	19.54	20.73	40.22	35.19	0.080
*Rhizobiaceae*	4.39	4.79	5.21	8.96	5.84	6.58	5.69	0.129
*Moraxellaceae*	1.10	1.71	1.67	0.71	0.64	0.69	1.32	0.519
*Pseudomonadaceae*	9.22a	15.98b	15.61bc	9.13 ac	7.55a	13.44abc	8.84a	0.007
*Caryophanaceae*	0.03a	0.07a	0.04ab	0.58b	0.04a	0.00a	0.02a	0.045
*Streptococcaceae*	0.47	1.46	0.33	0.52	1.02	0.51	0.74	0.624
Facultative anaerobic spore-formers^3^	0.51	1.57	1.11	0.68	1.09	0.63	0.51	0.116
Endophyte bacteria^4^	5.31	5.79	7.03	8.66	10.65	5.14	7.68	0.561
Rumen/fecal bacteria^5^	0.43	0.90	0.27	1.01	3.18	0.19	1.17	0.725
Potentially pathogenic bacteria^6^	1.79	0.18	0.30	0.51	2.78	0.17	0.21	0.301

1 Mean relative abundance of 16S rRNA V3-V4 amplicon taxonomic data of the top 12 most common bacterial families in grass-legume forage.

2 *p* values were generated by Kruskal-Wallis one-way non-parametric test at an alpha value of 0.05 and post-hoc pairwise comparisons were carried out with Dunn’s test. Lowercase letters represent comparison between farms where the same letter denotes a non-significant difference.

3 Facultative Anaerobic Spore Formers: *Bacillaceae, Paenibacillaceae.*

4 Endophytic bacteria: *Sphingomonadaceae, Sphingobacteriaceae, Xanthomonadaceae*.

5 Rumen/Fecal bacteria: *Lachnospiraceae, Ruminococcaceae*.

6 Potentially pathogenic bacteria (*Listeriaceae, Staphylococcaceae*).

### 3.2. Bacterial diversity of the silage

The alpha diversity (Shannon index) for corn silage (Table 4) showed a significant difference for farms only (L01 and L05 > all other farms; *p* = 0.004). For grass-legume silage (Table 4), there was a higher Shannon index for INOC vs. NIS (*p* = 0.029), and significant differences for farms (L05 > all other farms; *p* = 0.021) and seasons (no visible trends; *p* < 0.001).

**Table 4:** Significance (*p* values) of the effect of four factors on the Unweighted UniFrac (PERMANOVA) in corn and grass-legume silage.

Groups	Factors			
	Inoculation	Silo types	Farms	Seasons
Shannon index				
Corn	0.104	0.855	0.004	0.341
Grass	0.029	0.087	0.021	< 0.001
Unweighted unifrac				
Corn	0.125	0.005	0.001	0.001
Grass	0.007	0.001	0.001	0.001

PERMANOVA for unweighted Unifrac and Shannon index measures were carried out on the 16S rRNA phylogenetic data using Qiime2.

For corn silage, PERMANOVA for unweighted UniFrac on bacteria (Table 4) showed a significant difference between bunkers and towers (*p* = 0.005). Separation by farm showed a significant difference between farms (*p* < 0.001). Separation by sampling period showed a significant difference across each period.

Unweighted UniFrac of the bacterial community of the mixed grass-legume silage (Table 4) showed a significant difference between INOC and NIS (*p* = 0.007), between tower and bunker silo types (*p* < 0.001), among farm (*p* < 0.001) and across periods (*p* < 0.001).

### 3.3. Difference in bacterial population of silage across experimental factors

**Figure 1:**
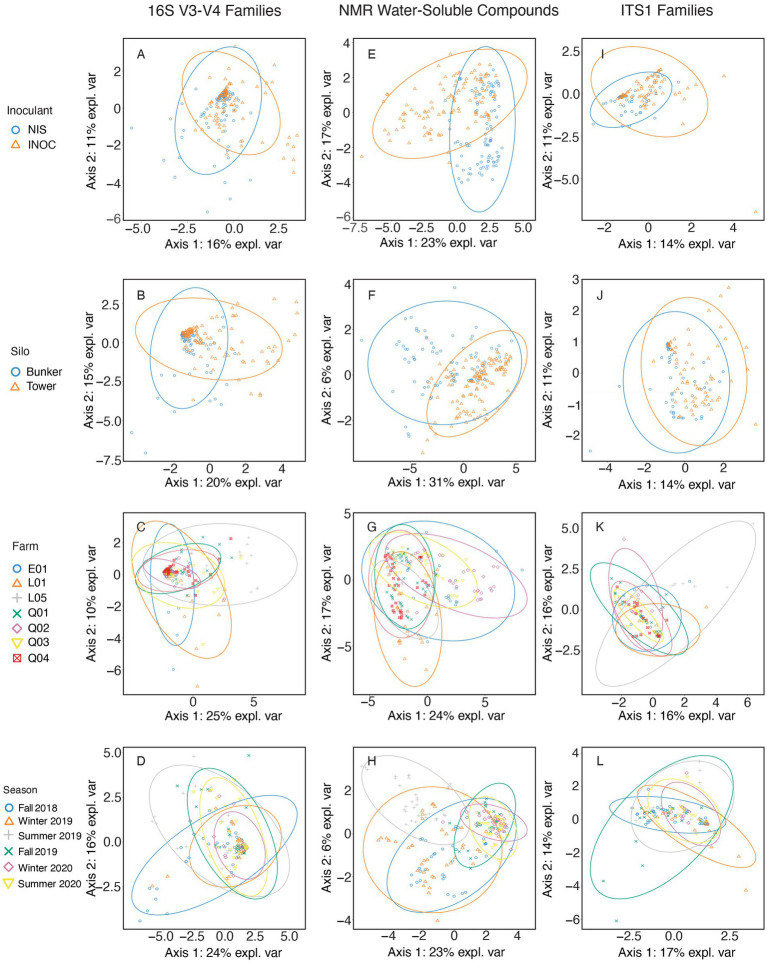
sPLS-DA of corn samples spanning all years and farms using 16S V3-V4 rRNA gene amplicon taxonomic data at a family level **(A–D)**, NMR metabolomic data in grams/kilogram of dry matter **(E–H)**, and ITS1 amplicon taxonomic data at a family level **(I–L)**. Samples are separated by either Inoculant **(A,E,I)**, Silo **(B,F,J)**, Farm **(C,G,K)**, or Season **(D,H,L)**.

The bacterial families in corn silage (Figure 1) separated by inoculation (Figure 1A) with the highest contribution to sPLS-DA loadings of NIS samples on axis 1 were the *Leuconostocaceae* (mean RA of 4.0% for NIS and 2.5% for INOC). This observation is confirmed by the *p* value obtained through Wilcoxon test (Table 5) for *Leuconostacaceae* depending on inoculation factor (*p* < 0.001). The families contributing to axis 2 were not significantly different between NIS and INOC.

**Table 5:** Significance (*p* values) of the effect of four factors on the variation in fermentation parameters, family level taxonomic relative abundance from 16S rRNA gene and ITS1 family level amplicon sequencing of the most abundant families, concentration of the main NMR compounds, and mycotoxins in corn silage.

Groups	Variables	Factors			
		Inoculation	Silo types	Farms	Seasons
Fermentation	pH	< 0.001	< 0.001	< 0.001	< 0.001
Bacteria	*Leuconostocaceae*	< 0.001	< 0.001	< 0.001	0.298
	*Lactobacillaceae*	0.834	0.219	< 0.001	0.004
	*Acetobacteriaceae*	0.741	0.943	0.091	< 0.001
	Facultative anaerobic spore-formers^1^	0.538	0.649	0.255	0.001
	*Clostridiaceae*	0.634	0.699	0.283	< 0.001
	*Enterobacteriaceae*	0.797	0.110	< 0.001	0.001
	*Rhizobiaceae*	0.575	0.003	0.006	< 0.001
	*Moraxellaceae*	0.009	0.402	< 0.001	< 0.001
	*Pseudomonadaceae*	0.186	0.214	< 0.001	< 0.001
	*Caryophanaceae*	0.932	0.001	< 0.001	< 0.001
	Endophyte bacteria^1^	0.577	0.009	0.256	< 0.001
	Shannon Index	0.104	0.855	0.004	0.341
Fungi	*Phaffomycetaceae*	0.878	0.020	0.022	0.008
	*Saccharomycetaceae*	0.777	0.009	< 0.001	< 0.001
	*Malasseziaceae*	0.261	0.002	0.002	0.002
	*Mucoraceae*	0.588	< 0.001	< 0.001	0.018
NMR	Acetate	< 0.001	< 0.001	< 0.001	0.111
compounds	Alanine	< 0.001	0.486	< 0.001	< 0.001
	Aspartate	0.431	0.008	< 0.001	0.004
	γ-aminobutyrate	< 0.001	0.023	< 0.001	0.347
	Propane-1,2-diol	< 0.001	< 0.001	< 0.001	0.113
	Citrate	0.351	0.178	0.176	0.554
	Glycerol	< 0.001	0.003	< 0.001	< 0.001
	Acetone	0.038	0.385	0.089	< 0.001
	Glycine	< 0.001	< 0.001	< 0.001	0.079
	Betaine	0.444	0.030	< 0.001	0.020
	Imidazole	0.844	0.054	0.007	< 0.001
	Valine	0.002	< 0.001	< 0.001	< 0.001
	Leucine	0.098	< 0.001	< 0.001	0.254
	Methanol	0.224	0.194	0.084	< 0.001
	Isoleucine	0.178	< 0.001	< 0.001	0.366
	Phenylalanine	0.058	< 0.001	< 0.001	0.121
	Mannitol	< 0.001	0.001	< 0.001	< 0.001
	Succinate	0.506	0.449	0.952	0.011
	Xylose	0.049	0.464	< 0.001	0.453
Mycotoxins	Fumonisin	0.051	0.253	< 0.001	0.163
	Fusaric acid	< 0.001	0.207	< 0.001	0.187
	Deoxynivalenol	0.886	0.252	< 0.001	0.045
	Zearalenone	0.261	0.309	< 0.001	0.005
	Beauvericin	0.043	0.427	0.001	0.078

^1^*p* values for 16S rRNA gene V3-V4 amplicon taxonomic relative abundance data (Bacteria), ITS1 amplicon taxonomic relative abundance data (Fungi), NMR metabolomic data (g/kg DM), and mycotoxin LC–MS data in corn samples were generated by Wilcoxon or Kruskal-Wallis one-way non-parametric test. Samples were grouped by Inoculant, Silo, Farm, and Season, and statistical analysis was done for each grouping separately. For Bacteria, fungi, and NMR, the significant families or compounds contributing to the sPLS-DA were chosen for this table. Some low abundance families were grouped as Facultative Anaerobic Spore Formers (*Bacillaceae, Paenibacillaceae*) and Endophytic bacteria (*Sphingomonadaceae, Sphingobacteriaceae, Xanthomonadaceae*). All mycotoxins measured in the mycotoxin extraction method used were chosen.

The families in samples separated by silo type (Figure 1B) with the highest contribution to loadings of tower silo samples on axis 1 were *Leuconostocaceae* (mean RA of 4.3% for towers and 1.5% for bunkers, *p* < 0.001 Table 5), *Caryophanaceae* (0.8% for towers and 0.3% for bunkers, *p* = 0.001 Table 5), *Rhizobiaceae* (0.2% for towers and nd for bunkers, *p* = 0.003, Table 5), and plant related bacterial families (0.6% for towers and 0.2% for bunkers, *p* = 0.009, Table 5). No families contributing to axis 2 had significant differences through the Wilcoxon test.

The families separated by farm (Figure 1C) with the highest contribution to loadings of farm L05 samples across axis 1 were *Caryophanaceae* (mean RA of 1.9% for L05 and 0.4% for other farms; Supplementary Table S1) and *Moraxellaceae* (5.5% for L05 and 0.7% for other farms), and for farm Q02 were *Lactobacillaceae* (88.6% for farm Q02 and 77.4% for other farms). Families with the highest contribution to loadings of farm L01 samples on axis 2 were *Enterobacteriaceae* (4.5% for farm L01 and 0.9% for all other farms). Contribution to loadings of farm Q02 samples across axis 2 were *Lactobacillaceae*, and for farm L05 were *Leuconostocaceae* (5.9% for farm L05 and 2.7% for other farms). The *p* values for all these families RA among farms were < 0.001 (Table 5).

The family separated by sampling period (Figure 1D) with the highest contribution to loadings of Summer 2019 samples across axis 1 was the *Acetobacteraceae* (4.5% for Summer 2019 and 1.8% for other seasons). Contributions to loadings of Fall 2018 samples across axis 1 were the grouping of families for facultative anaerobic spore formers (7.8% for Fall 2018 and 1.5% for other seasons) and *Caryophanaceae* (1.4% for Fall 2018 and 0.4% for other seasons). The family with the highest contribution to loadings of Fall 2018 samples across axis 2 was *Clostridiaceae* (0.7% for Fall 2018 and 0.2% for other seasons). For Summer 2018 samples across axis 2, families with the highest contributions to loadings were the families grouped as plant-based bacteria (1.0% for Summer 2018 and 0.2% for other seasons), and *Moraxellaceae* (2.1% for Summer 2018 and 1.2% for other seasons). The *p* values for all these families RA across seasons were < 0.001 (Table 5).

**Figure 2:**
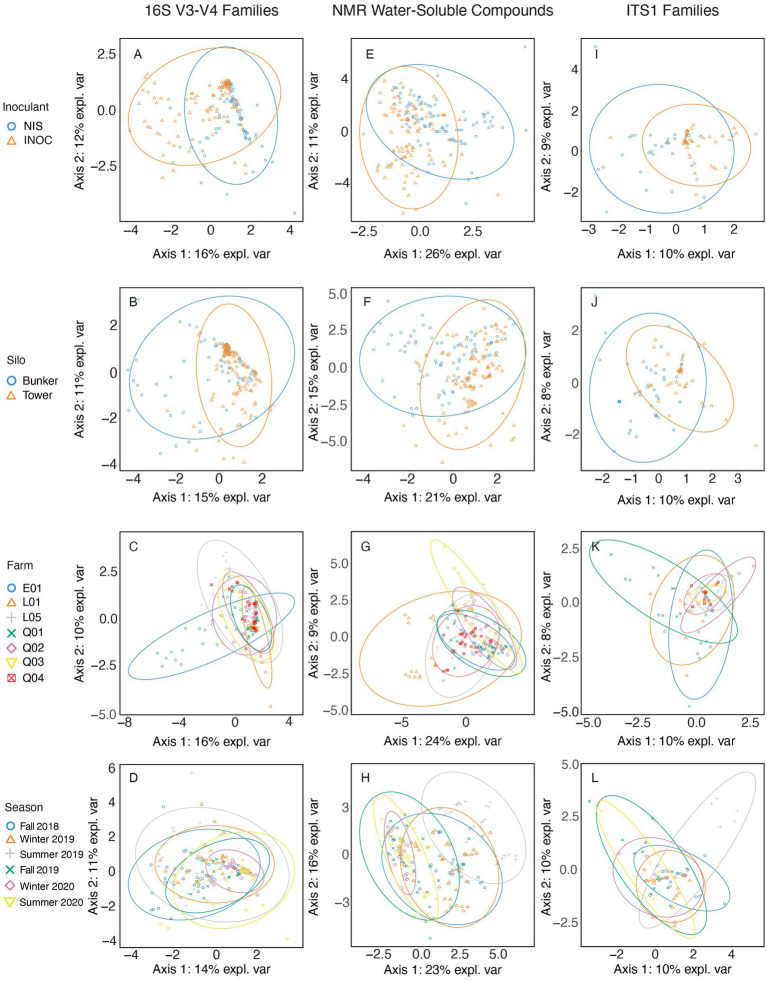
sPLS-DA of mixed grass-legume samples spanning all years and farms using 16S V3-V4 rRNA gene amplicon taxonomic data at a family level **(A–D)**, NMR metabolomic data in grams/kilogram of dry matter **(E–H)**, and ITS1 amplicon taxonomic data at a family level **(I–L)**. Samples are separated by either Inoculant **(A,E,I)**, Silo **(B,F,J)**, Farm **(C,G,K)**, or Season **(D,H,L)**.

The bacterial families from mixed grass-legume silage (Figures 2A–D) with the highest contribution to sPLS-DA loadings of NIS samples on axis 1 (Figure 2A) were *Leuconostocaceae* (mean RA of 15.6% for NIS and 8.4% for INOC, *p* < 0.01, Table 6), and *Lactobacillaceae* (70.0*%* for NIS and 56.3% for INOC, *p* = 0.005, Table 6). The family with the highest contribution to loadings of INOC samples across axis 1 was *Caryophanaceae* (0.7% for INOC and 0.3% for NIS, *p* = 0.006, Table 6). Families with the highest contribution to loadings of INOC samples across axis 2 were *Pseudomonadaceae* (0.9% for INOC and 0.2% for NIS, *p* = 0.012, Table 6) and *Planoccocaceae*.

**Table 6:** Significance (*p* values) of the effect of four factors on the variation in fermentation parameters, family level taxonomic relative abundance from 16S rRNA gene and ITS1 family level amplicon sequencing of the most abundant families, concentration of the main NMR compounds, and mycotoxins in grass-legume silage.

Groups	Variables	Factors			
		Inoculation	Silo types	Farms	Seasons
Fermentation	pH	< 0.001	0.031	< 0.001	0.947
Bacteria	*Leuconostocaceae*	< 0.001	0.030	< 0.001	< 0.001
	*Lactobacillaceae*	0.005	0.684	< 0.001	< 0.001
	Facultative anaerobic spore-formers	0.071	0.445	0.362	0.366
	*Clostridiaceae*	0.706	0.978	< 0.001	0.711
	*Pseudomonadaceae*	0.012	0.852	0.009	0.598
	*Caryophanaceae*	0.006	0.601	< 0.001	< 0.001
	Rumen/fecal bacteria	0.062	0.287	0.006	0.457
	Shannon index	0.029	0.087	0.021	< 0.001
Fungi	*Phaffomycetaceae*	0.045	0.764	0.062	0.008
	*Saccharomycetaceae*	0.451	0.019	0.198	0.002
	*Malasseziaceae*	0.043	0.482	0.131	< 0.001
	*Mucoraceae*	0.516	0.559	0.488	0.414
NMR	Acetate	0.342	0.435	0.038	< 0.001
	γ-aminobutyrate	0.543	0.997	0.168	0.345
	Propane-1,2-diol	0.575	0.619	< 0.001	0.085
	Aspartate	< 0.001	0.017	< 0.001	< 0.001
	Asparagine	0.436	0.202	< 0.001	< 0.001
	Dimethylamine	0.519	0.859	0.445	< 0.001
	Glycerol	0.994	0.199	0.002	0.647
	Glycolate	< 0.001	0.730	0.012	0.002
	Hypoxanthine	0.585	0.035	0.153	0.333
	Acetone	0.786	0.222	0.017	< 0.001
	Butyrate	0.376	0.054	0.003	< 0.001
	Betaine	0.056	< 0.001	< 0.001	< 0.001
	Ethanol	0.987	0.218	0.732	< 0.001
	Leucine	0.441	0.149	0.021	0.137
	Phosphocholine	0.065	0.112	0.003	0.433
	Phenylalanine	0.424	0.649	< 0.001	0.201
	Imidazole	0.842	0.120	< 0.001	< 0.001
	Tyramine	0.031	0.072	0.024	< 0.001
	Arabinose	0.035	0.717	< 0.001	< 0.001
	Galactose	0.566	0.225	< 0.001	< 0.001
	Xylose	0.001	0.276	0.002	0.237
Mycotoxins	Fusaric acid	0.361	0.414	0.576	0.572
	Deoxynivalenol	0.207	0.881	0.154	0.459
	Zearalenone	0.259	0.515	0.673	0.057
	Beauvericin	0.550	0.057	0.163	0.086
	Alternariol	0.003	0.996	0.017	0.012

*p* values for 16S rRNA gene V3-V4 amplicon taxonomic relative abundance data (Bacteria), ITS1 amplicon taxonomic relative abundance data (Fungi), NMR metabolomic data (g/kg DM), and mycotoxin LC–MS data in grass-legume samples were generated by Wilcoxon test or Kruskal-Wallis one-way non-parametric test. Samples were grouped by Inoculant, Silo, Farm, and Season, and statistical analysis was done by each grouping separately. For Bacteria, fungi, and NMR, the significant families or compounds contributing to the sPLS-DA were chosen for this table. Some low abundance families were grouped as Facultative Anaerobic Spore Formers (*Bacillaceae, Paenibacillaceae*) and Rumen/Fecal bacteria *(Lachnospiraceae, Ruminococcaceae*). All mycotoxins measured in the mycotoxin extraction method used were chosen.

The family in grass-legume separated by silo (Figure 2B) with the highest contribution to loadings of tower silo samples across axis 1 was *Leuconostocaceae* (mean RA of 13.6% for towers and 9.2% for bunkers, *p* = 0.03, Table 6).

The families in mixed grass-legume silage separated by farm (Figure 2C) with the highest contribution to loadings of farm E01 samples across axis 1 were *Clostridiaceae* (mean RA of 3.7% for farm E01 and 0.3% for other farms, *p* < 0.001, Supplementary Table S2) and *Caryophanaceae* (1.2% for farm E01 and 0.2% for other farms, *p* < 0.001, Table 6) and for farm L01 was *Lactobacillaceae* (77.6% for farm L01 and 59.6% for other farms, *p* < 0.001, Table 6). The family with the highest contribution to loadings of farm E01 samples across axis 2 was the grouping of rumen-based bacteria (3.0% for farm E01 and 1.7% for other farms, *p* = 0.006, Table 6).

The family in mixed grass-legume silage separated by season (Figure 2D) with the highest contribution to loadings of Winter 2020 samples across axis 1 was *Lactobacillaceae* (72.9% for Winter 2020 and 61.2% for other seasons, *p* < 0.001, Table 5) and for Fall 2018 was *Leuconostocaceae* (16.5% for Fall 2018 and 10.5% for other seasons, *p* < 0.001, Table 6). There were no families with significant differences that contributed to axis 2.

### 3.4. Correlations between pH, dry matter, and bacterial families

The correlation matrix between pH, dry matter, and bacterial families in corn silage (Supplementary Figure S1) showed positive correlations between dry matter and pH, and a negative correlation between dry matter and *Peptostreptococcaceae*, *Bifidobacteriaceae*, *Prevotellaceae*, and *Listeriaceae*. The pH was positively correlated with *Leuconostocaceae*, *Brevibacteriaceae*, *Burkholderiaceae*, *Clostridiaceae*, *Bifidobacteriaceae*, and *Prevotellaceae*.

The correlation matrix between pH, dry matter, and bacterial families in grass-legume silage (Supplementary Figure S2) showed a positive correlation between dry matter and *Acetobacteraceae*, *Lachnospiraceae*, and *Bifidobacteriaceae*. The pH was positively correlated with *Leuconostocaceae* only.

### 3.5. Discriminant analysis of NMR compounds from silage

The NMR compounds in corn silage (Figures 1E–H) separated by inoculation (Figure 1E) with the highest contribution to sPLS-DA loadings of INOC samples were glycerol (mean concentration of 2.3 g/kg DM for INOC and 1.7 g/kg DM for NIS), propane-1,2-diol (7.0 g/kg DM for INOC and 1.4 g/kg DM for NIS), γ-aminobutyrate (1.6 g/kg DM for INOC and 1.1 g/kg DM for NIS), and acetate (15.5 g/kg DM for INOC and 8.5 g/kg DM for NIS). The compound with the highest contribution to loadings of NIS samples across axis 1 was mannitol (9.7 g/kg DM for NIS and 4.2 g/kg DM for INOC). All compounds presented *p* values <0.001 between inoculation factors (Table 5).

The compounds in corn silage separated by silo type (Figure 1F) with the highest contribution to loadings of bunker silo samples across axis 1 were leucine (3.0 g/kg DM for bunkers and 2.0 g/kg DM for towers, *p* < 0.001, Table 5), and isoleucine (1.2 g/kg DM for bunkers and 0.8 g/kg DM for towers, *p* < 0.001, Table 5). The compounds with the highest contribution to loadings of bunker silo samples across axis 2 was betaine (0.4 g/kg DM for bunkers and 0.3 g/kg DM for towers, *p* = 0.030, Table 5) and contribution to loadings of tower silo samples across axis 2 was valine (1.5 g/kg DM for towers and 1.1 g/kg DM for bunkers, *p* < 0.001, Table 5).

Compounds in corn silage separated by farm (Figure 1G) with the highest contribution to loadings of farm Q02 samples across axis 1 were glycine (mean concentration of 1.7 g/kg DM for Q02 and 0.5 g/kg DM for other farms), propane-1,2-diol (13.3 g/kg DM for farm Q02 and 2.0 g/kg DM for other farms), acetate (19.5 g/kg DM for farm Q02 and 10.9 g/kg DM for other farms), and γ-aminobutyrate (2.0 g/kg DM for farm Q02 and 1.3 g/kg DM for other farms). All compounds presented *p* values <0.001 among farms (Table 5). There were no significant differences in chemical compounds contributing to axis 2.

Compounds in corn silage separated by sampling periods (Figure 1H) with the highest contribution to loadings of Summer 2019 samples across axis 1 were acetone (2.1 g/kg DM for Summer 2019 and 0.4 g/kg DM for other seasons) and imidazole (0.6 g/kg DM for Summer 2019 and 0.4 g/kg DM for other seasons). All compounds presented *p* values <0.001 across seasons (Table 5).

The NMR compound in mixed grass-legume silage (Figures 2E–H) separated by inoculant (Figure 2E) with the highest contribution to sPLS-DA loadings of NIS samples across axis 1 was xylose (mean concentration of 0.7 g/kg DM for NIS and 0.4 g/kg DM for INOC, *p* = 0.001; Table 6). There were no significant differences for chemical compounds contributing to axis 2.

The compound in grass-legume silage separated by silo type (Figure 2F) with the highest contribution to loadings of bunker silo samples across axis 1 was betaine (mean concentration of 1.0 g/kg DM for bunkers and 0.6 g/kg DM for towers, *p* < 0.001, Table 6). There were no significant compound contributing to tower silos across axis 2.

The compound in mixed grass-legume silage separated by farm (Figure 2G) with the highest contribution to loadings of L01 samples across axis 1 was aspartate (mean concentration of 5.4 g/kg DM for farm L01 and 2.1 g/kg DM for other farms; Supplementary Table S2). Compounds with the highest contribution to loadings of farm Q03 samples across axis 2 were propane-1,2-diol (4.5 g/kg DM for farm Q01 and 1.6 g/kg DM for other farms) and asparagine (3.2 g/kg DM for farm Q03 and 2.0 g/kg DM for other farms). All compounds presented *p* values <0.001 among farms (Table 5).

Compounds in mixed grass-legume silage separated by sampling periods (Figure 2H) with the highest contribution to loadings of Summer 2019 samples across axis 1 were acetone (2.1 g/kg DM for Summer 2019 and 0.7 g/kg DM for other seasons) and ethanol (7.2 g/kg DM for Summer 2019 and 3.2 g/kg DM for other seasons). Compounds with the highest contribution to loadings of Fall 2018 samples across axis 2 were arabinose (1.0 g/kg DM for Fall 2018 and 0.6 g/kg DM for other farms) and galactose (1.6 g/kg DM for Fall 2018 and 1.1 g/kg DM for other seasons). All compounds presented *p* values <0.001 across seasons (Table 6).

### 3.6. Compositional variation in the fungal community of silage

There were no significant differences in any fungal family in the sPLS-DA of corn silage separated by inoculation (Figure 1I).

The family in corn silage separated by silo type (Figure 1J) with the highest contribution to loadings of tower silo samples across axis 1 was *Mucoraceae* (mean RA of 4.8% for towers and 2.7% for bunkers, *p* < 0.001, Table 5) and for bunkers across axis 1 was *Malasseziaceae* (6.0% for bunkers and 2.1% for towers, *p* = 0.002; Table 5).

Fungal families of the corn silage separated by farm (Figure 1K) with the highest contribution to loadings of farm L05 samples across axis 1 was *Phaffomycetaceae* (mean RA of 14.0% for farm L05 and 2.27% for other farms, *p* = 0.020, Supplementary Table S1) and for farm L01 was *Saccharomycetaceae* (70.68% for farm L01 and 40.5% for other farms, *p* = 0.009, Table 5). The family with the highest contribution to loadings of farm L05 samples across axis 2 was *Phaffomycetaceae*. The family with the highest contribution to loadings of farm L01 samples across axis 2 was *Saccharomycetaceae*, and for farm Q01 across axis 2 was *Mucoraceae* (7.5% for farm Q01 and 3.5% for other farms, *p* < 0.001, Table 5).

Fungal families of the corn silage separated by season (Figure 1L) with the highest contribution to loadings of Winter 2020 samples across axis 1 was *Saccharomycetaceae* (mean RA of 65.9% for Winter 2020 and 38.5% for other seasons, *p* < 0.001, Table 5), and for Winter 2019 across axis 1 was *Malasseziaceae* (4.9% for Winter 2019 and 3.1% for other seasons, *p* = 0.002, Table 5). The family with the highest contribution to loadings of Summer 2019 samples across axis 2 was *Phaffomycetaceae* (1.8% for Summer 2019 and 0.3% for other seasons, *p* = 0.022, Table 5).

The fungal family in mixed grass-legume silage (Figures 2I–L) separated by inoculation (Figure 2I) with the highest contribution to sPLS-DA loadings on axis 1 was *Phaffomycetaceae* (mean RA of 5.7% for NIS and 1.6% for INOC, *p* = 0.045, Table 6). The fungal family of the mixed grass-legume silage by silo (Figure 2J) with the highest contribution to loadings of bunker samples across axis 1 was *Saccharomycetaceae* (mean RA of 32.5% for bunkers and 16.7% for silos, *p* = 0.019, Table 6). There were no significant differences in fungal families of the mixed grass-legume silage separated by farm (Figure 2K).

### 3.7. Quantification of mycotoxins in silage

Mycotoxins in corn silage (Table 5) analyzed by inoculant showed a higher concentration of beauvericin (mean concentration of 342.1 mg/kg DM for INOC and 223.2 mg/kg DM for NIS; *p =* 0.043), fumonisin (166.7 mg/kg DM for INOC and 88.7 mg/kg DM for NIS; *p =* 0.051), and fusaric acid (1212.2 mg/kg DM for INOC and 437.4 mg/kg DM for NIS; *p <* 0.001) content in INOC over NIS. There were no significant differences when separated by silo type. Separation by farm shows a lower concentration of DON (mean concentration of 161.5 mg/kg DM for farm Q02 and 587.3 mg/kg DM for other farms; *p <* 0.001) and zearalenone (0.7 mg/kg DM for farm Q02 and 27.0 mg/kg DM for other farms; *p <* 0.001) in Q02 compared to all other farms. The concentration of fumonisin (261.1 mg/kg DM for farm E01 and 103.0 mg/kg DM for other farms; *p* < 0.001) and fusaric acid (2325.2 mg/kg DM for farm E01 and 620.9 mg/kg DM for other farms; *p* < 0.001) were higher in silage from farm E01 than all other farms. Separation by period shows a higher concentration of DON (mean concentration of 3730.0 mg/kg DM for Fall 2018 and 372.4 for other seasons; *p <* 0.001) and zearalenone (195.5 mg/kg DM for Fall 2018 and 11.1 mg/kg DM for other seasons; *p <* 0.001) in Fall 2018 than all other periods.

In mixed grass-legume silage, there was a higher concentration of alternariol in NIS compared to INOC (mean concentration of 14.1 mg/kg DM for NIS and 4.4 mg/kg DM for INOC; *p =* 0.018).

### 3.8. Variation in composition of the microbial community and NMR compounds in milk

**Figure 3:**
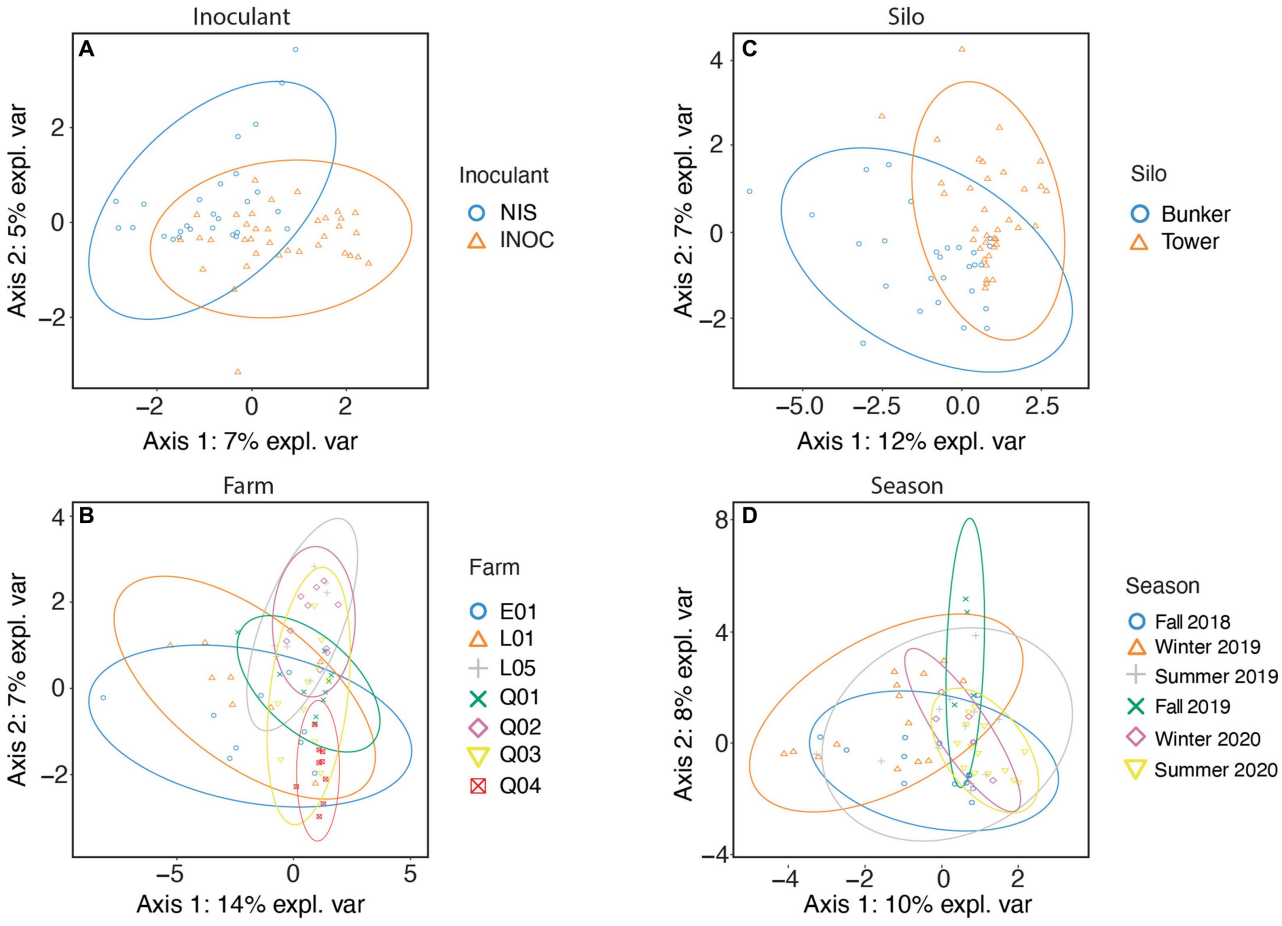
sPLS-DA of milk samples spanning all years and farms using 16S rRNA gene V3-V4 amplicon taxonomic data at a family level. Samples are separated by either Inoculant **(A)**, Farm **(B)**, Silo **(C)**, or Season **(D)**.

The bacterial family in milk (Figure 3) separated by inoculation of silage (Figure 3A) with the highest contribution to sPLS-DA loadings of NIS samples on axis 1 (7% explained variance) was *Staphylococcaceae* (mean RA of 10.2% for NIS and 4.9% for INOC; Table 7).

**Table 7:** Significance (*p* values) of the effect of four factors on the variation in family level taxonomic relative abundance from 16S rRNA gene family level amplicon sequencing of the main contributing families to sPLS-DAs in bulk tank milk.

Groups	Variables	Factors			
		Inoculation	Silo types	Farms	Seasons
Bacteria	*Leuconostocaceae*	0.066	0.162	0.028	0.539
	*Lactobacillaceae*	0.074	0.505	< 0.001	0.245
	*Paenibacillaceae*	0.189	0.007	0.092	< 0.001
	*Moraxellaceae*	0.038	0.545	0.013	0.125
	*Pseudomonadaceae*	0.366	0.388	0.054	0.079
	*Xanthomonadaceae*	0.737	0.498	0.699	< 0.001
	*Erysipelotrichaceae*	0.749	0.005	0.009	0.238
	*Staphylococcaceae*	0.025	0.033	0.001	0.205

*p* values for 16S rRNA gene V3-V4 amplicon taxonomic relative abundance data (Bacteria) were generated by Wilcoxon test or Kruskal-Wallis one-way non-parametric test. Samples were grouped by Inoculant, Silo, Farm, and Season, and statistical analysis was done by each grouping separately. All families were analyzed using the sPLS-DA method, however only the significant families that were relevant to sPLS-DA axis contributions are reported in this table.

The bacterial family in milk (Figure 3C) with the highest contribution to loadings of bunker silo samples on axis 1 was *Erysipelotrichaceae* (mean RA of 3.1% for bunkers and 1.1% for towers, *p* = 0.005, Table 7) and for tower samples across axis 1 was *Paenibacillaceae* (11.6% for towers and 4.1% for bunkers, *p* = 0.007, Table 7).

The bacterial families in milk separated by farm (Figure 3B) with the highest contribution to loadings of farm L05 samples across axis 2 were *Moraxellaceae* (11.1% for farm L05 and 3.8% for other farms, *p* = 0.013, Supplementary Table S3) and (20.1% for farm L05 and 3.5% for other farms, *p* = 0.054, Table 7) and for farm Q04 across axis 2 was *Staphylococcaceae* (mean RA of 21.6% for farm Q04 and 4.7% for other farms).

The bacterial family in milk (Figure 3D) with the highest contribution to loadings of Winter 2019 samples across axis 1 were *Xanthomonadaceae* (mean RA of 13.3% for Winter 2019 and 3.2% for other seasons, *p* < 0.001, Table 7) and for Fall 2019 across axis 1 was *Paenibacillaceae* (15.0% for Fall 2019 and 9.2 for other seasons, *p* < 0.001 Table 7).

## 4. Discussion

### 4.1. Diversity of forage bacteria

The epiphytic microbial profile of forage is affected by farm management practices and environmental conditions such as temperature and humidity variations, organic fertilization or soil contamination during harvesting. Nazar et al. (2022) showed that the diversity of the epiphytic microbial community differs between the type of forage (grasses, legumes), but also between species within a type, such as within the grasses (napier and sudan grass). The farms that we studied did indeed use different types of grass (timothy, brome, canary, orchard, fescue, festulolium) which could explain part of the variation in relative abundance of certain bacterial families (*Lactobacillaceae*, *Pseudomonadaceae*, and *Caryophanaceae*). No studies have been published on the difference in diversity of the epiphytic microbiota on the specific grasses and mixtures of grasses used in our study. Due to the high overlap between single grass types across multiple farms, we were unable to observe any key findings in the microbiota of farm silage based on individual grass types. Furthermore, Nazar et al. (2022) showed that, although there were important differences in beta diversity of the epiphytic microbiota between grasses, a short ensiling period of 3 days reduced this difference.

The use of fertilizers affects forage microbiota, as the addition of manure instead of mineral fertilizers can change the microbial diversity of forages, including increased clostridial counts during fermentation (Müller et al., 2014; Drouin et al., 2022). In our study, the *Clostridiaceae* may have been affected by the use of liquid manure fertilizer, as the only farm to use strictly liquid manure (farm E01) had a higher RA of *Clostridiaceae* and rumen-based bacteria in grass-legume silage. However, this trend was not observed before the forage was ensiled and was not observed in corn forage or silage from this farm.

### 4.2. Variation in microbial diversity and metabolome of corn silage

Silo type is an important but understudied aspect of farm management that could have an important role in shaping the microbiota of silage. In corn silage, the RA of *Leuconostocaceae* was higher in tower silos than in bunker silos. *Leuconostocaceae* are not adapted to the low pH encountered in low buffering capacity silage, so the abundance of bacteria from this family is generally low in corn silage, which often has a lower pH compared to grass-legume silage (Zhang et al., 2017). In the current study, the higher RA of *Leuconostocaceae* was correlated with higher pH in the corn silage samples and grass-legume, which aligned with the higher *Leuconostocaceae* in towers compared to bunkers in corn silage. This higher pH can be due to a lower density common to silage from tower silos, specifically closer to the upper section (Muck et al., 2015) that was collected, which could explain why the *Leuconostocaceae* are higher in tower silos of corn silage. Furthermore, the higher pH and lower density attributed to tower silos may have led to a higher RA of *Saccharomycetaceae. Saccharomycetaceae* are common in corn silage and generally dominate the fungal community, particularly in the presence of oxygen which favors yeast growth (Drouin et al., 2021). Although our study did not count yeast cells, it is likely that tower silos had higher total yeast count as tower silos are associated with a higher abundance of yeast (McCallum, 2020) likely due to the increased oxygen from lower density silage (Muck et al., 2015).

Leucine and isoleucine, particularly in corn silage, were higher in bunker silos. These compounds can contribute to pre-weaning growth in early life of dairy calves, which can have long term beneficial effects on milk production (Reiners et al., 2022). To our knowledge, there is no literature explaining the variation of leucine and isoleucine levels in silage. However, Park et al. (2017) compared leucine catabolism between two *Lactobacillaceae* and *Leuconostocaceae* isolates from kimchi and determined that the *Lactobacillaceae* isolates produced more leucine than the *Leuconostocaceae*, particularly at a lower pH. Although Park et al. (2017) studied isolates instead of the microbial community, it is possible that the higher RA of the *Leuconostocaceae* and higher pH in tower silos would be linked to the lower leucine and isoleucine in corn silage.

Due to the restricted geographic utilization and the security challenges posed for sampling tower silos (Bahloul et al., 2012), less reliable details on the fermentation parameters of this type of silo are available, although they are recognized as having lower DM losses (Wilkinson and Rinne, 2018). More studies are required to describe the microbial populations and metabolome of this silo type.

The use of the lactic acid bacteria species *L. buchneri* as an inoculant has been shown to increase RA of *Lactobacillaceae* in silage compared to NIS and to reduce *Leuconostocaceae* (Romero et al., 2018). For both corn and grass-legume silage, *L. buchneri* inoculants have been shown to increase the concentrations of acetic acid, propane-1,2-diol, propionic acid, and γ-aminobutyrate. In our study, the farms that used an inoculant had significantly higher levels of these compounds in corn silage than in silage from farms not using a microbial silage additive. However, on a farm-to-farm basis, mainly farms Q02 and Q03 (located in the same geographical area of Montérégie, Québec), showed higher content of all four of those compounds alongside higher RA of *Lactobacillaceae* and lower RA of *Leuconostocaceae*. This highlights the differences due to either specific farm practices or region. A regional difference in the microbial community of corn silage was previously shown in Iran by Gharechahi et al. (2017). The community diversity (alpha diversity) was shown to be higher in the farms from Southwestern Ontario (L01 and L05) than the other farms. This may point to a potential need for a higher inoculation rate for plant material from some farms due to a more diverse natural microbiota of corn, as RA of *Lactobacillaceae* was lower in these farms after ensiling compared to other farms.

Other factors that affect inoculation success include the size of the inoculant population compared to the epiphytic population, which could have antagonistic activity toward successful fermentation (Kurkjian et al., 2021). It is therefore important for farmers to closely adhere to the application methods provided by inoculant companies to achieve the desired efficacy, particularly in relation to the rate of application, as they have been strictly optimized to obtain ideal performance from each of the bacterial species.

Farm management has a strong impact on the overall quality of silage. Silage from farm Q02 showed a very typical bacterial community and metabolome for silage that has been inoculated. Silage from farm Q02 had higher *Lactobacillaceae* than other farms, a trend that has been observed both with inoculation of *L. plantarum* and *L. buchneri* (Han et al., 2022), and even more so as fermentation time increases (da Silva et al., 2021). The higher amounts of γ-aminobutyrate, 1,2-propanediol, and acetate in silage from farm Q02 are also strong indicators of a successful fermentation and high quality silage (Arriola et al., 2021). Furthermore, farm Q02 showed a lower amount of deoxynivalenol and zearalenone in corn silage, suggesting lower fungal activity or the successful destruction of mycotoxins during fermentation (Ogunade et al., 2018). The indicators of a successful fermentation were not visible in all inoculated silage from farms, however, and some NIS farms (Q01 in particular) showed indicators of good fermentation, such as high *Lactobacillaceae*, 1,2-propanediol, propionate, and acetate in grass-legume silage. This indicates that regional epiphytic bacteria could produce a high-quality silage with proper fermentation.

The metabolite profile of silage shows a very clear separation of Summer 2019 samples from the other seasons. The amount of acetone was higher in Summer 2019 than all other seasons. Ethanol and butyrate were also higher, but only in grass-legume silage. Generally, a high concentration of butyrate in silage is an indicator of clostridial fermentation (Ávila and Carvalho, 2020). Acetone and ethanol are also associated with clostridial fermentation from the acetone-butanol-ethanol pathway (Luo et al., 2016). Interestingly, the relative abundance of *Clostridiaceae* in grass-legume silage from the summer of 2019 was not higher than for the other seasons, even though the metabolites seem to indicate clostridial activity. This is likely due to the presence of DNA from dead or sporulated *Clostridium* cells, which may mask the variation in live cultivable populations. This trend was not visible in the 2020 summer season, which may indicate a higher temperature in the weeks prior to the 2019 summer sampling period. Borreani and Tabacco (2010) showed that temperature differences between 25 and 35°C showed significant increases in butyrate content and clostridia at each ascending temperature interval in corn silage.

Studies suggest that corn is much more susceptible to mycotoxin contamination than other forage types (Křížová et al., 2021). The toxins beauvericin, fumonisin, and fusaric acid are all indicators of common silage molds, particularly *Fusarium*, often present in silage (Shimshoni et al., 2013). The LAB inoculant species *L. buchneri* does not reduce mycotoxins produced by common silage molds (Ogunade et al., 2018), while inoculants *L. plantarum* and *P. pentosaceus* have been associated with higher levels of DON in corn silage in a trial by Wang et al. (2018). The inoculants used in the silage in our study did not have any effect on mycotoxin limitation with some mycotoxins even reaching higher levels in INOC corn silage. The mechanisms behind the increased mycotoxin content of inoculated corn silage are unclear but may be indirectly due to preservation of the mycotoxins because of lower temperatures of the silage core through reduction of aerobic activity brought on by the use of an inoculant (Wang et al., 2018). That being said, farm Q02 (successful silage fermentation) had lower mycotoxins than other farms. The inoculated silage from this farm had a community profile and metabolome of corn and grass-legume silage that could be associated with inoculation (Arriola et al., 2021). This could indicate that regional effects or proper use of an inoculant could reduce the concentrations of mycotoxin in inoculated silage (Ogunade et al., 2018).

Seasonal effects on the bacterial diversity at the family taxonomic level following fermentation of corn silage were much less impactful than farm or region, but there was a visible trend with samples from Fall 2018 (grouping September and November). Samples from the fall period generally include corn silage harvested the previous year and stored for around 12 months. Fall samples presented higher RA of facultative anaerobic spore formers (*Bacillus* and *Paenibacillus*) than all other seasons. Growth and presence of these bacteria are associated with higher pH and oxygen content (Ávila and Carvalho, 2020), conditions associated with the early stage of fermentation or the outer area of bunker silos. Since our sample collection started at silage from the previous year, the presence of these spore formers is likely because of repeated exposure to oxygen and gradual increase in pH over a full year of bunker opening and feed-out. The DON and zearalenone contents were significantly higher in the Fall 2018 season than all other seasons. Corn silage feed-out from the fall generally comes from silage that was put into silos the previous year. The production of DON and zearalenone is produced by field mold, and the mycotoxins are shown to increase over time during ensiling (Jensen et al., 2020). This may explain the high levels of DON during fall seasons. The high level of these two mycotoxins observed only in Fall 2018 line up with other aerobic activity or markers of poor-quality silage of these samples, such as the growth of facultative anaerobic and strict anaerobic spore formers. This indicates that conditions for ensiling were less ideal in the year prior to the trial, reflected in lower quality silage for Fall 2018, but not for Fall 2019.

### 4.3. Variation in the microbial diversity and metabolome of grass-legume silage

Important differences in the management of the harvest frequency and the type of fertilizer applied during the growing season were present between the farms selected for this study. These factors influenced the microbiota profiles and their evolution over time. Most of the farms applied mainly mineral based fertilizer during the growing season, but one of the farms used liquid manure. The grass-legume silage from this farm presented the 2^nd^ highest RA from families that can be grouped as rumen/fecal related bacteria in our study, the higher RA being observed for another farm using liquid manure and mineral as fertilizers. Specific rumen families have been shown to concentrate in manure, including *Ruminococcaceae*, *Lachnospiraceae*, *Rikanellaceae*, and *Clostridiaceae* (Ozbayram et al., 2018). This link may be evident in grass-legume silage because the rumen/fecal bacteria are generally sensitive to low pH (Li et al., 2021). and grass-legume silage has a higher pH in comparison to corn silage.

Muck et al. (2015) showed that the use of a bunker silo compared to a tower silo could result in lower DM and higher clostridial fermentation of alfalfa silage. Our study has shown that there is a difference in the microbial diversity of towers and bunker silos, but the absence of a difference in the RA of the *Clostridiaceae* between the two silo types does not point to any advantage of using tower or bunker silos. The higher RA of *Clostridiaceae* in grass-legume silage from farm E01 (bunker silo) suggests that the combination of liquid manure as fertilizer, together with the use of a bunker silo, may be the cause of increased *Clostridiaceae* in silage samples (Müller et al., 2014; Muck et al., 2015). As mentioned for the corn silage, the volume of lower quality silage in bunker silos is observed mainly below the plastic cover and the side walls while the complete surface of the feed-out zone of tower silos could be at a lower density and prone to deterioration, specifically the upper section of the silo (Spörndly, 2018).

The use of an inoculant by some of the farms was clearly visible in corn silage, particularly through the metabolome. However, this trend was not as clear in mixed grass-legume silage. There were significantly less *Lactobacillaceae* in inoculated silage compared to non-inoculated except for one of the farms in the Montérégie region, and there were no significant differences in the contents of acetic acid, propionic acid, and of γ-aminobutyrate. Furthermore, the microbial diversity was not classified by farm region as seen for corn silage. There was, however, the same trend in propane-1,2-diol in grass-legume as with corn, which was numerically higher in the inoculated farms of Montérégie than all other farms. Da Silva et al. (2017) showed that inoculation with a strain of *L. buchneri* was correlated with higher propane-1,2-diol but no correlation was observed with acetic acid content. The presence of higher RA of some genera from the *Leuconostocaceae*, which can also produce acetic acid (Paillart et al., 2016) could potentially have higher metabolic impact in the first months of ensiling over the strain of *L. buchneri* inoculated on the forage. Furthermore, the RA of *Lactobacillaceae* does not equate to the relative abundance of the inoculant strain of *L. buchneri* (Drouin et al., 2021), so it is possible that the RA of *Lactobacillaceae* observed in the mixed grass-legume silage was not necessarily a direct indicator of inoculant efficacy. We did, however, also observe a relationship between inoculation and higher RA of the *Lactobacillaceae* in grass-legume silage. Overall, the specific farm or region seems to have a more pronounced effect on the bacterial community and the NMR metabolome of corn silage than it does on mixed grass-legume silage.

The inoculated samples of grass-legume silage from farm E01, which was stored in a bunker, had higher levels of *Caryophanaceae* (formerly *Planococcaceae*). *Lysinibacillus* is a genus of *Caryophanaceae* that is commonly found in silage and indeed represented close to 100% of the *Caryophanaceae* in our silage samples. The *Lysinibacillus* may be associated with late fermented silage (Wang et al., 2022) as well as silage that has been exposed to air (Liu et al., 2013; Duniere et al., 2017). Although the *Caryophanaceae* may be an indicator of aerobically compromised silage, there is no known negative or positive side effects of higher abundance of this bacterial family in silage or milk.

Alternariol was the only mycotoxin that showed a higher amount in NIS compared to INOC with grass-legume silage. Alternariol is produced by species from the fungal genus *Alternaria*, which are described as field fungi common in grass forages (Cheli et al., 2013). Alternariol is a genotoxic (Miao et al., 2022) mycotoxin (observed on bacterial and mammalian cells *in vitro*), however little is known about its *in vivo* effects on dairy cattle (Gallo et al., 2015). The inoculation of grass-legume silages may prevent the accumulation of alternariol, although more research must be done into the specific effects of alternariol on dairy cattle.

### 4.4. Microbiota of raw milk

*Staphylococcus* in milk is associated both with human infection primarily from raw milk cheeses (Rola et al., 2016), and mastitis in dairy cows (Adesiyun et al., 1998). In the current study, the *Staphylococcaceae* were lower in raw milk from farms that used INOC compared to NIS. Several genera from the *Lactobacillaceae* family have been shown to inhibit the growth of *Staphylococcus aureus* in milk. The suggested mechanisms for inhibitory antimicrobial effects by *Lactobacillaceae* against *Staphylococcus aureus* are the production of bacteriocins, hydrogen peroxide, lactic acid, and acetic acid (Karska-Wysocki et al., 2010). However, no link between the RA of *Staphylococcaceae* and *Lactobacillaceae* in milk was observed. Moreover, our study did not identify any correlation between *Lactobacillaceae* in silage and *Staphylocaccaceae* in milk, thus the potential impact of silage inoculants on milk microbiota remains inconclusive. It may very well be that the farms with the practice of inoculation also had a lower incidence of clinical mastitis in their herd.

Spore formers observed in silage have also been detected in raw milk (te Giffel et al., 2002). Our study shows a higher abundance of *Paenibacillaceae* in raw milk from farms that used tower silos. However, there was no difference in facultative anaerobic spore formers (*Bacillaceae*, *Paenibacillaceae*) by silo type in either corn or grass-legume silage.

The RA of *Moraxellaceae* was higher in raw milk from farms L05 and Q02. *Acinetobacter* is the dominant genus from the family *Moraxellaceae* in silage studies (Bai et al., 2021) and was the main genus of *Moraxellaceae* ASVs in both silage and milk in our study. *Acinetobacter* are occasionally pathogenic (Towner, 2009), and can cause lipolysis in milk, causing spoilage through rancidity and off-flavors (Hantsis-Zacharov and Halpern, 2007). The RA of *Moraxellaceae* in the corn silage of farm L05 was higher than all other farms, and it was higher in the grass-legume silage of Q02 compared to all other farms. This points to the possible transfer of *Moraxellaceae* from both corn and grass-legume silage to the milk, which is then collected in the bulk tank. However, no correlation was observed between the *Moraxellaceae* family from silage and milk.

The effects of season on milk microbiota were less pronounced than farm management practices. The presence of *Xanthomonadaceae* was higher during the winter of 2020 which was also observed by Celano et al. (2022). In their study, Celano et al. (2022) correlated the higher presence of *Xanthomonadaceae* with *Enterobacteriaceae* and the bovine ketosis marker β-hydroxybutyrate in milk samples. They suggested that higher *Xanthomonadaceae* and *Enterobacteriaceae* may represent a shift towards a microbial niche that leads to lower milk quality, however our study did not observe any significant differences in *Enterobacteriaceae*. On its own, there is no evidence that *Xanthomonadaceae* could be linked to bovine ketosis or lower milk quality. In the winter seasons, the RA of *Xanthomonadaceae* was higher (but not significant) in NIS than in INOC, suggesting that the use of an inoculant on silage may contribute to lowering the relative abundance of *Xanthomonadaceae* in raw milk.

## 5. Conclusion

Season, farm, silo type, and inoculation clearly affected the chemical variation of both corn and grass-legume silage, while the family level microbiota was not as clear. Individual farm practices may have obscured and dampened some of the observed effects of season and inoculation, suggesting that farm management practices and location may be equally as important. The use of an inoculant was not able to fully control the variations in silage microbiota as we see in mini-silos or from single farm experiments. In this study we did not observe changes in silage microbiota that were correlated with variations in milk microbiota although it is likely that silage management practices including use of an inoculant, silo type, and farm geographical location can affect the microbiota of bulk tank raw milk, but more studies must be done to confirm and expand upon these observations.

## Data availability statement

The data presented in this study are deposited in the Borealis repository, and is accessible through this link: https://borealisdata.ca/dataset.xhtml?persistentId=doi:10.5683/SP3/C8CZBK.

## Author contributions

PD and GL contributed to the conception and experimental design of the study, project administration and obtained the funding. JH collected and analyzed the samples, performed statistical analysis, prepared the figures, and wrote the first draft. PD organized the database and generated the metadata sheet. JR performed the mycotoxin analysis and wrote the related materials and methods section. PD, GL, and LD reviewed and edited the manuscript. All authors contributed to the article and approved the submitted version.

## Funding

Funding for this study was provided by the NSERC Collaborative Research and Development Grant (CRDPJ 529498–18) as well as the NSERC/DFO Industrial Research Chair in Dairy Microbiology (490979–15) held by GL.

## Conflict of interest

JH, PD, JR, and GL declare that the research was conducted in the absence of any commercial or financial relationships that could be construed as a potential conflict of interest. LD is employed by Lallemand Inc., however, their affiliation did not impede their ability to follow journal guidelines or to remain impartial during the preparation of this manuscript.

## Publisher’s note

All claims expressed in this article are solely those of the authors and do not necessarily represent those of their affiliated organizations, or those of the publisher, the editors and the reviewers. Any product that may be evaluated in this article, or claim that may be made by its manufacturer, is not guaranteed or endorsed by the publisher.

## Footnotes

^1^ www.mixomics.org/methods/splsda/
