# Supplementary material for: Farm management practices and season dependent factors affect the microbial community and chemical profile of corn and grass-legume silages of farms in Ontario, Québec, and Northern New York
Source: Front Microbiol. 2023 Jul 19;14:1214915. doi: 10.3389/fmicb.2023.1214915 (PMC10394519; doi:10.3389/fmicb.2023.1214915)
Supplement: Supplementary file 1 [file Data_Sheet_1.PDF]

Supplementary Table S1. Relative abundance (%) of the most common bacterial and fungal families from 16S and ITS1 amplicon sequencing; concentrations of NMR water-soluble compounds (g/kg DM); and concentrations of mycotoxins (µg/kg DM) in the corn silage separated by farm.

Groups	Variables	Farms							
		E01	L01	L05	Q01	Q02	Q03	Q04	*P* [1]
Bacteria	*Leuconostocaceae*	1.49 [2]	2.14	5.85	4.98	0.88	2.40	4.53	< 0.001
	Lactobacillaceae	80.12	68.61	55.43	84.15	88.64	82.27	81.41	< 0.001
	Acetobacteriaceae	1.45	5.76	4.22	2.81	1.96	3.07	2.47	0.091
	Facultative anaerobic spore-formers	0.62	2.28	7.08	1.34	1.56	2.71	0.94	0.255
	Clostridiaceae	0.20	0.34	0.38	0.34	0.34	0.17	0.31	0.283
	Enterobacteriaceae	2.39	4.53	2.87	1.20	0.55	0.82	1.32	< 0.001
	Rhizobiaceae	0.10	0.09	0.48	0.11	0.10	0.23	0.11	0.006
	Pseudomonadaceae	1.58	0.59	0.68	1.14	0.44	0.06	0.31	< 0.001
	Caryophanaceae	0.13	0.29	1.85	0.59	0.52	0.51	0.49	< 0.001
	Moraxellaceae	0.23	2.48	5.45	0.12	0.85	0.45	0.50	< 0.001
	Endophyte bacteria	0.14	0.19	1.00	0.55	0.15	0.16	0.53	0.256
	Rumen/fecal bacteria	0.50	1.81	1.36	0.51	0.76	1.65	0.50	0.378
	Potentially pathogenic bacteria	4.46	1.92	0.23	0.16	0.04	1.27	1.10	0.380
Fungi	*Phaffomycetaceae*	3.67 [3]	1.32	13.95	4.14	2.64	0.71	1.14	0.022
	Saccharomycetaceae	31.61	70.68	65.28	43.28	23.60	35.44	43.64	< 0.001
	Malasseziaceae	3.23	4.20	BT [4]	0.27	11.90	6.47	BT	0.002
	Mucoraceae	1.55	0.35	3.61	7.46	6.93	3.65	4.79	< 0.001
NMR (g/kg DM)	Acetate	18.49 [5]	9.04	6.66	8.36	19.53	14.54	8.17	< 0.001
	Lactate	39.26	52.62	50.10	33.91	41.32	33.88	45.86	< 0.001
	γ-amino butyric acid	1.48	1.03	1.21	1.16	2.00	1.63	1.03	< 0.001
	Propane-1,2-diol	4.31	0.73	0.47	1.93	13.32	7.29	1.17	< 0.001
	Propionate	1.10	0.75	2.11	0.86	0.87	0.80	1.74	0.176
	Glycerol	2.04	2.19	1.13	1.30	2.70	2.22	1.60	< 0.001
	Acetone	0.82	1.23	1.13	0.88	0.89	0.64	0.86	0.089
	Butyrate	2.55	0.43	0.60	0.59	1.77	0.56	0.37	< 0.001
	Betaine	0.29	0.32	0.30	0.30	0.37	0.57	0.43	< 0.001
	Ethanol	8.17	5.58	2.53	4.33	5.29	3.56	5.59	< 0.001
	Leucine	2.52	3.21	1.93	1.95	3.27	2.18	2.00	< 0.001
	Methanol	0.28	0.35	0.53	0.46	0.32	0.32	0.44	0.084
	Isoleucine	1.11	1.20	0.78	0.86	1.21	0.85	0.79	< 0.001
	Xylose	1.48	2.69	2.66	1.29	1.05	1.24	1.75	< 0.001

Mycotoxin	Fumonisin	261.11 [6]	ND [7]	96.17	ND	93.13	197.06	231.72	< 0.001
(µg/kg DM)	Fusaric acid	2325.20	273.00	439.00	60.05	914.47	1100.13	938.94	< 0.001
	Deoxynivalenol	511.40	1221.11	642.83	317.35	161.47	453.17	377.56	< 0.001
	Zearalenone	37.60	52.67	24.67	7.35	0.73	24.06	15.39	< 0.001
	Beauvericin	499.60	0.00	53.33	59.30	448.47	262.11	516.94	< 0.001

[1] *P* values were generated by Kruskal-Wallis one-way non-parametric test at an alpha value of 0.05.

[2] Mean relative abundance of 16S V3-V4 amplicon taxonomic data of the top 12 most common bacterial families in corn silage.

[3] Mean relative abundance of ITS amplicon taxonomic data of the top 5 most common bacterial families in corn silage

[4] BT: Below Threshold; Relative abundance below the cut-off of 0.1%

[5] Mean concentrations (µg/kg DM) of the top 5 most common LC-MS generated mycotoxins in corn silage

[6] Mean concentrations (g/kg DM) of the top 14 most common NMR water soluble compounds in corn silage

[7] ND: Not Detectable

Supplementary Table S2. Relative abundance (%) of the most common bacterial and fungal families from 16S and ITS1 amplicon sequencing; concentrations of NMR water-soluble compounds (g/kg DM); and concentrations of mycotoxins (µg/kg DM) in the grass-legume silage separated by farm.

Groups	Variables	Farms							
		E01	L01	L05	Q01	Q02	Q03	Q04	*P* [1]
Bacteria	*Leuconostocaceae*	6.92 [2]	10.58	13.91	15.67	10.21	3.29	2.38	< 0.001
	Lactobacillaceae	43.86	77.57	47.54	70.60	64.48	68.81	62.20	< 0.001
	Acetobacteriaceae	0.11	0.15	BT [3]	1.36	1.07	BT	0.12	< 0.001
	Facultative anaerobic spore-formers	3.45	0.39	0.23	4.49	5.26	5.67	3.57	0.362
	Clostridiaceae	3.68	BT	0.16	0.26	0.29	BT	0.10	< 0.001
	Enterobacteriaceae	1.10	2.09	1.68	0.89	1.43	1.86	0.70	0.382
	Rhizobiaceae	0.25	0.93	0.69	0.62	0.24	0.27	0.25	0.009
	Pseudomonadaceae	1.38	0.24	0.39	BT	0.94	0.78	0.28	0.009
	Caryophanaceae	1.22	BT	0.82	0.35	0.56	0.24	0.49	< 0.001
	Moraxellaceae	0.54	1.13	1.25	BT	2.22	0.11	1.50	< 0.001
	Endophyte bacteria	0.77	0.24	0.23	0.24	0.18	0.24	0.33	0.859
	Rumen/fecal bacteria	2.96	3.99	1.18	1.40	1.48	1.02	1.13	0.006
	Potentially pathogenic bacteria	1.74	0.38	4.67	0.15	2.01	1.73	1.25	0.137
Fungi	*Phaffomycetaceae*	4.12 [4]	8.64	0.48	6.79	0.25	0.10	1.68	0.062
	Saccharomycetaceae	28.32	43.95	24.26	20.31	26.88	11.71	10.48	0.198
	Malasseziaceae	4.38	4.20	BT	8.57	0.10	BT	0.10	0.131
	Mucoraceae	6.84	2.77	6.34	8.64	7.41	1.00	3.86	0.488
NMR (g/kg DM)	Acetate	0.40 [5]	12.45	12.32	14.51	16.13	10.74	12.43	0.038
	Lactate	57.12	37.17	55.48	39.55	44.72	34.04	49.60	< 0.001
	γ-amino butyric acid	2.59	3.56	3.63	3.27	3.73	3.19	3.21	0.168
	Propane-1,2-diol	0.29	2.13	1.13	1.48	3.43	4.51	1.29	< 0.001
	Propionate	0.85	0.59	0.54	1.42	0.95	1.17	2.38	0.002
	Glycerol	3.13	3.68	2.72	2.42	2.59	3.48	3.06	0.002
	Acetone	0.62	1.25	0.94	0.63	1.28	0.94	0.87	0.017
	Butyrate	1.04	0.98	1.64	1.16	1.64	0.76	0.54	0.003
	Betaine	1.36	0.87	0.49	0.76	0.93	0.60	0.50	< 0.001
	Ethanol	2.84	3.74	4.43	2.71	3.59	4.85	3.00	0.732
	Leucine	4.37	5.41	4.37	3.63	4.29	4.15	4.73	0.021
	Methanol	1.02	1.58	0.71	1.06	0.97	1.01	1.18	0.003
	Isoleucine	2.64	3.61	2.76	2.46	2.52	2.43	2.78	0.016
	Xylose	0.58	0.84	0.43	0.63	0.39	0.40	0.54	0.002

Mycotoxins (µg/kg DM)	Fumonisin	ND [6]	ND	ND	ND	ND	ND	ND	N/A
	Fusaric acid	ND	ND	10.31	ND	ND	14.57	ND	0.576
	Deoxynivalenol	155.18 [7]	52.90	ND	78.89	1.53	25.13	47.20	0.459
	Zearalenone	ND	ND	ND	1.39	ND	ND	2.33	0.057
	Beauvericin	ND	ND	ND	17.78	ND	19.19	ND	0.086
	Alternariol	11.27	10.80	4.20	11.61	4.23	ND	19.33	0.017

[1] *P* values were generated by Kruskal-Wallis one-way non-parametric test at an alpha value of 0.05

[2] Mean relative abundance of 16S V3-V4 amplicon taxonomic data of the top 12 most common bacterial families in grass-legume silage.

[3] BT: Below Threshold; Relative abundance below the cut-off of 0.1%

[4] Mean relative abundance of ITS amplicon taxonomic data of the top 5 most common bacterial families in grass-legume silage

[5] Mean concentrations (g/kg DM) of the top 14 most common NMR water soluble compounds in grass-legume silage

[6] ND: Not Detected

[7] Mean concentrations (µg/kg DM) of the top 5 most common LC-MS generated mycotoxins in grass-legume silage

Supplementary Table S3. Relative abundance (%) of the most common bacterial and fungal families from 16S and ITS1 amplicon sequencing in the raw bulk tank milk

Variables	Farms (Relative abundance % and *P* value)							
	E01	L01	L05	Q01	Q02	Q03	Q04	*P* [1]
Leuconostocaceae	0.60 [2]	1.61	0.10	1.74	0.29	1.22	2.15	0.028
Lactobacillaceae	1.44	2.37	0.30	1.29	14.19	14.51	1.68	< 0.001
Bacillaceae	1.12	3.04	2.75	3.93	1.04	2.21	2.27	0.745
Paenibacillaceae	6.80	3.78	8.69	16.13	1.89	8.58	11.89	0.097
Clostridiaceae	0.66	0.48	0.39	0.38	0.10	0.47	0.16	0.653
Enterobacteriaceae	9.55	8.46	2.18	4.75	1.86	3.31	1.24	0.457
Rhizobiaceae	0.00	0.18	0.13	0.00	0.16	0.37	0.38	0.001
Pseudomonadaceae	1.64	4.11	17.77	0.48	9.52	4.18	1.17	0.054
Caryophanaceae	0.13	BT [3]	BT	0.11	0.39	BT	0.13	0.945
Xanthomonadaceae	10.51	7.02	1.53	2.65	0.94	8.12	10.53	0.689
Moraxellaceae	1.10	3.53	13.99	3.36	8.82	4.49	1.30	0.025
Streptococcaceae	4.49	1.60	8.56	28.02	13.94	3.60	2.37	0.027
Enterococcaceae	0.50	0.58	0.10	0.81	3.00	0.59	0.32	0.153
Staphylococcaceae	8.17	4.48	2.17	4.42	0.96	7.61	21.71	0.001
Listeriaceae	4.30	0.60	1.01	5.15	0.10	1.46	2.06	0.001

[1] *P* values were generated by Kruskal-Wallis one-way non-parametric test at an alpha value of 0.05

[2] Mean relative abundance of 16S V3-V4 amplicon taxonomic data of the top 15 most common bacterial families in raw bulk tank milk.

[3] BT: Below Threshold; Relative abundance below the cut-off of 0.1%

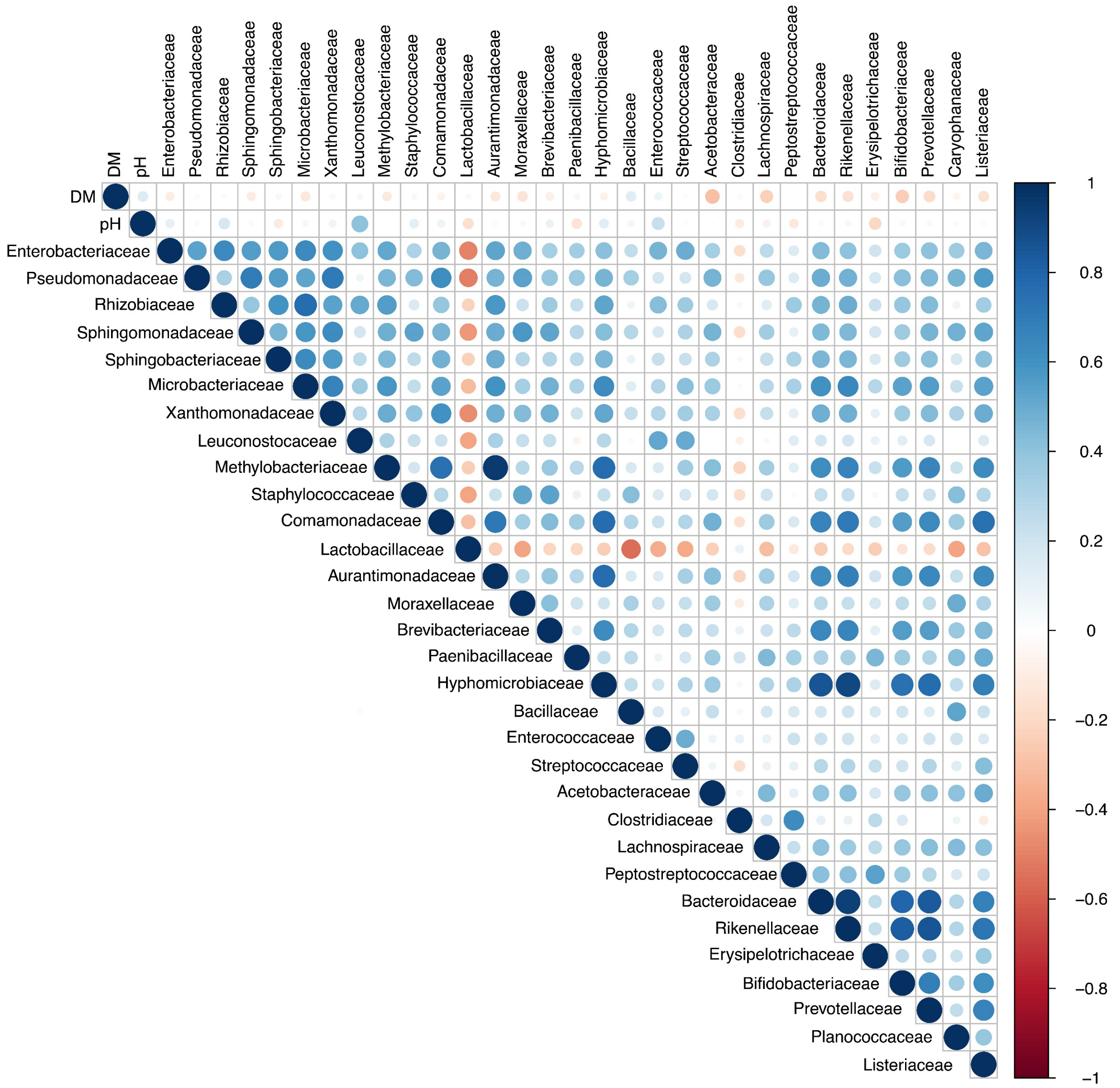

Supplementary Figure S1. Correlation matrix between family level 16S V3-V4 rRNA gene amplicons, pH, and dry matter content (DM) in corn silage across all farms.

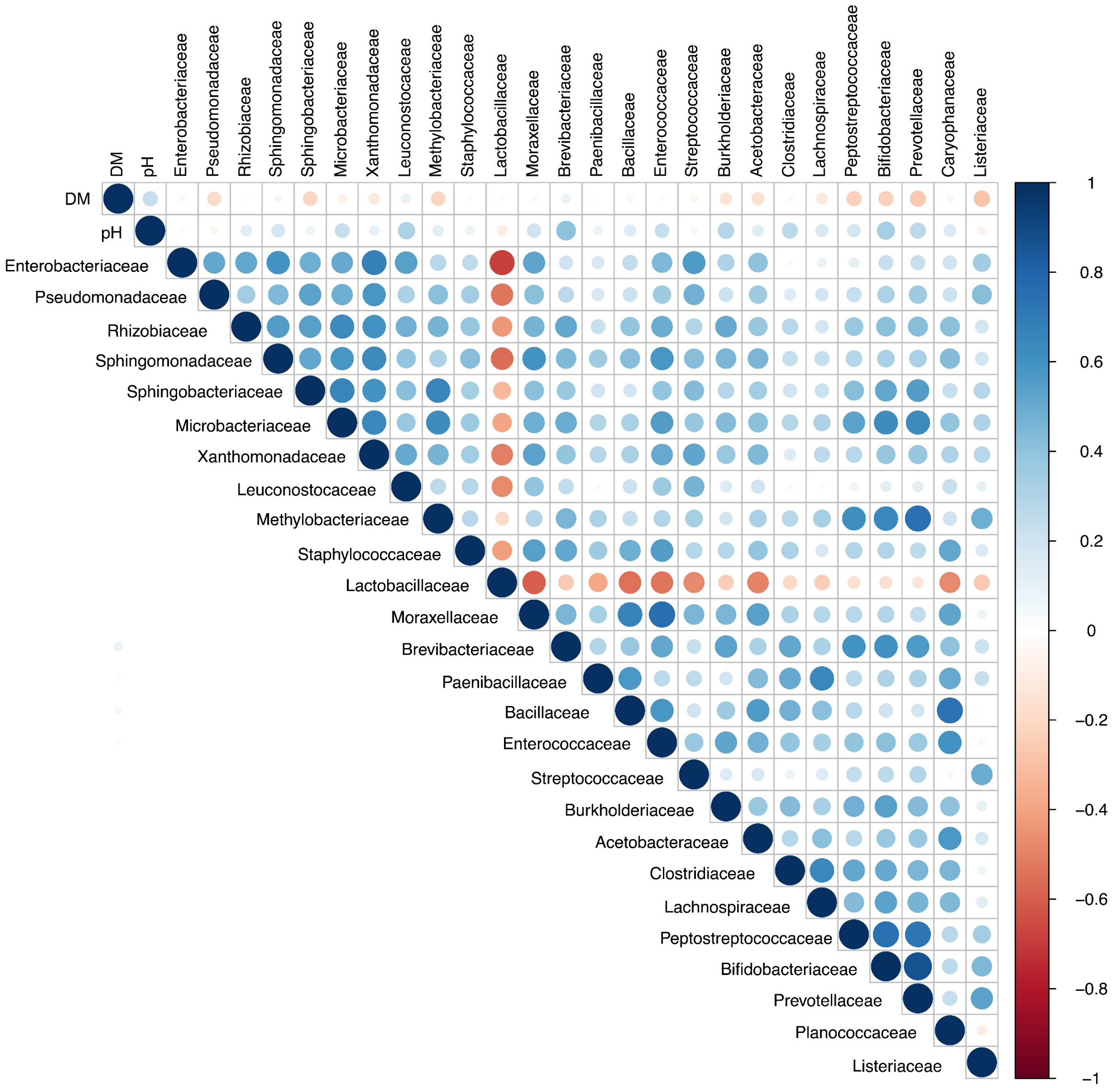

Supplementary Figure S2. Correlation matrix between family level 16S V3-V4 rRNA gene amplicons, pH, and dry matter content (DM) in grass-legume silage across all farms.
